# Fifty Thousand Years of Extinction

**DOI:** 10.1371/journal.pbio.1001186

**Published:** 2011-11-01

**Authors:** Adrian Lister

**Affiliations:** The Natural History Museum, London, United Kingdom

## Abstract

Adrian Lister reviews *Once and Future Giants*.

**Figure pbio-1001186-g001:**
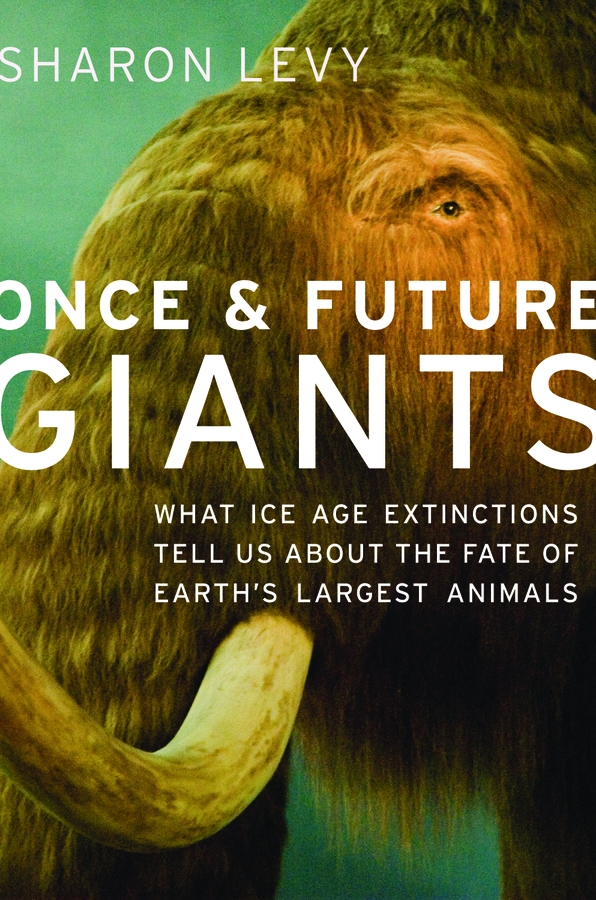
Levy S (2011) Once and Future Giants: What Ice Age Extinctions Tell Us About the Fate of Earth's Largest Animals. Oxford University Press USA. 280 p. ISBN-13: 978-0195370126 (hardcover). $US24.95.

[Fig pbio-1001186-g001]The Late Quaternary extinction of megafauna has been a “hot topic” of research and debate since at least the time of Darwin (who discussed it in the *Origin*), but especially since the 1960s when Paul Martin ignited the debate with his theory of prehistoric overkill. The topic has had increasing exposure over the past ten years because of the relevance of this, the most recent palaeontological extinction event, to modern concerns about biodiversity loss. Although sometimes described as a “mass extinction”, the Late Quaternary event, by itself, does not compare with the major extinctions of the distant past, such as the Late Permian event, when an estimated 90% of species went extinct, or the Cretaceous-Tertiary (K/T) event, with 75%, both decimating multiple groups of animals and plants across the marine, terrestrial, and freshwater biomes. The Late Quaternary event, by contrast, affected only large mammals (generally defined as >44 kg adult body mass), so even with the major losses in this group (around 70% of American species and 90% of Australia's), the number of species lost was less than a thousand, of the order of 0.01% of total global biodiversity.

Yet, as Sharon Levy convincingly argues in her excellent book, *Once and Future Giants*, the impact of this event, at least from a human perspective, is much greater than such figures would suggest. The ecological effect on many terrestrial ecosystems has been profound, the lost biomass being replaced by humans and their domestic livestock. Some authors go further and suggest that the megafaunal extinction, now known to have played out over the past 40,000 years, represents the opening act of an ongoing, anthropogenic decimation of the biosphere that may build into a mass extinction to rival those of the geological past.

The basic outline of the debate has changed little in the past 50 years, although we now have far more data and a variety of sophisticated tools. Martin's key insight, that extinction followed the spread of humans across the globe, remains broadly intact: Australia first, some time around 50–40,000 years ago, then the Americas, between about 14–12,000 years ago, and in the last 2,000 years, the faunas of islands such as Madagascar, New Zealand, and the West Indies as they became colonised by humans. Yet as extinctions are studied in more detail, with the aid of radiocarbon dating and other means, we see that the picture is more complex. Opponents of Martin's view had long cited climate change, during the last ice age and in the transition to the present interglacial, as key to the extinctions, and detailed mapping of climatic and vegetational change shows that mammal species did respond with major range shifts and often severe contractions that threatened their survival. It is also clear that different species' extinctions were not always synchronous in a given region, in ways that probably relate to their individual ecologies. Yet, as overkill proponents have always pointed out, previous ice age climatic cycles did not result in major extinctions. Recent years have seen a growing, though not universal, consensus that to some extent unites these perspectives: the human role in extinction may have been critical, but only because large-mammal populations were reduced, stressed, and fragmented by the effects of natural climate and vegetation change.

Levy deals with all of these issues in a highly readable and insightful way. She has interviewed many of the key researchers, especially in North America and Australia, and gives an accurate, up-to-date, and well-referenced account of their work. Proxies range from spores of dung-feeding fungi as a marker for megafauna, mammoth-tusk-rings for estimating life-history variables, computer-modelling of prehistoric human hunting, and ancient DNA to estimate changing past population sizes. Her approach is refreshingly even-handed, explaining the strengths and weaknesses of each model without an evident agenda to favour one theory over another.

The other great strength of the book is that it provides, in a more convincing way than I have read anywhere else, an integrated account of the past extinction of megafauna, the impact of these losses on the modern world, and the present status and conservation of large mammals globally. A rather moving passage describes the influence of Australian Aboriginals on the megafauna, at first possibly contributory to extinctions, but more recently maintaining ecosystems and biodiversity through their land-management practices, now being replaced by arguably more damaging official policy. On the other side of the world, the effects of commercial hunting and global warming on Arctic megafauna such as whales and reindeer are powerfully brought home in relation to the effects not only on the species themselves, but also on the native peoples who formerly (sustainably) exploited them.

Discussed in a no less sensitive and balanced manner are the more recent proposals for “re-wilding” of regions depauperate of large mammals due to Late Quaternary extinctions and more recent human intervention. Almost no ecosystems on Earth are truly “wild” in the sense of being unaffected by human activity, and we have to work with this situation, including the coexistence of native and introduced species, in conserving the natural world. Experiments in northern Siberia and Greenland, reintroducing horses and musk-oxen into their former ranges, indicate that grazing can help to convert wet tundra habitats into drier, grassy terrain more akin to that of the Late Pleistocene, and may help to mitigate the effects of climate warming in the erosion of permafrost environments. Much more controversial is the proposed introduction into the Great Plains of North America of exotic species such as Old World elephants and camels to “replace” analogous (at least confamilial) species such as mammoths and North American camelids lost to extinction. Levy draws on the chequered history of intentionally or non-intentionally introduced alien species such as the dingo and dromedary in Australia, or feral mustang horses in North America, whose control can pose major problems because they are not part of a balanced ecological community. Yet the introduction of unfenced populations of large predators such as lions into the North American interior is acknowledged to be politically unrealistic.

Even in these days of cloning and ancient DNA, we will not regain the megafauna we have lost. But understanding the causes of that loss and its impact on our world, as well as striving to retain the large mammals that remain, are crucial, as Levy argues convincingly and without hyperbole, to our own survival as a species.

**About the Author**

Adrian M. Lister is a Merit Researcher in Palaeontology at the Natural History Museum in London. His research is centred on the large mammals of the Quaternary, especially elephants and deer, and spans both fossil and modern representatives. He is particularly interested in Quaternary mammals as models for exploring patterns of speciation, adaptive evolution, and extinction. Professor Lister is the author of *Mammoths: Giants of the Ice Age* (3rd edition 2007, with Paul Bahn) and *Evolution on Planet Earth: The Impact of the Physical Environment* (2003, with Lynn Rothschild).

